# A Case of Purpura Hemorrhagica

**Published:** 1848-01

**Authors:** Samuel B. Robison

**Affiliations:** Rutherford county, Tennessee


Art. II.—*A Case of Purpura Hemorrhagica.* By Samuel B. Robison, M.D., of Rutherford county, Tennessee.

The following case is reported, not because it affords any thing new, either in pathology or treatment; but because of the rareness of the disease, and the singularity of its phases. On the 7th of last August I was called to see the child of Mr. Robert H. Wood, of this vicinity, aged about fourteen months, and found it laboring, as I thought, under the ordinary bowel complaint of the season, for which I prescribed some astringent powders, such as I usually give in such cases, composed of calomel, prepared chalk, acetate of lead, and sulphate of morphia, in minute portions. About night I was called again, when I found it presenting the train of symptoms that opium usually induces; but in addition to these, there was a singular trembling and partial protrusion of the tongue. As the diarrhœa had ceased suddenly from the opiate powder, I gave it an enema, but without relief. I next gave it an emetic of ipecac, which relieved it of the distress it then seemed to labor under, and it fell into a sweet sleep and rested well the remainder of the night. On my return next day, I found that its bowels had again become disordered, and that the body of the child was covered nearly all over with rubeolous patches which, declining after a few hours upon one part of the body, reappeared upon another, until every inch of the surface seemed to be visited by the eruption. In a day or two, a small jet black spot, not much larger than a flea bite, made its appearance upon the right side of the neck, about one inch from the *pomum adami*, and upon examination I discovered similar specks over the abdomen. One appeared upon the tip of the tongue, and about this time the tongue became very sore, and the mercurial fetor was perceived in its breath. The gums were not swollen or tender. After a few days the general soreness of the tongue subsided, but upon this, a tumor, of the size of the end of a man's thumb, made its appearance under the tongue on the left side, of a livid color, and the consistency of the liver. This substance remained for about two weeks, and was finally thrown off and discharged per anum, leaving the tongue red and raw. About the time of the appearance of this unnatural substance under the tongue, blood was discharged from the mouth in considerable quantities, coming apparently from the fauces, and very shortly afterwards from the penis also in even larger quantities. These discharges were kept up for some time. Its bowels had now become regular, and the stools of a healthy character. Appetite generally very good. The black spots now faded away, to be replaced by dark colored blotches, as if the skin had been bruised by external violence. These remained until the child died. Blood was discharged from all the external orifices in quantities sufficient, it appeared to me, to drain the system; but still the strength of the patient did not visibly decline. Appetite remained good, but the emaciation became extreme. On the 27th of September, fifty-one days from the time I first saw it, the child died, apparently from hemorrhage from the throat.

Our treatment in this case, we must confess, was purely experimental. We tried spirits of turpentine, muriatic and nitro-muriatic acid, mur. tinct. iron, cathartics, sarsaparilla, quinine, and some domestic remedies. One seemed to do about as much good as another, and none seemed to arrest in the slightest degree the fatal progress of the disease.

October 14, 1847.

